# Change of serum prealbumin levels and serum protein markers between egg white powder and casein protein additives in standard enteral feeding formulas in critically ill patients with acute respiratory failure

**DOI:** 10.1186/s40560-016-0157-0

**Published:** 2016-04-27

**Authors:** Rungsun Bhurayanontachai, Sunatee Sa-nguansai

**Affiliations:** Division of Critical Care Medicine, Department of Internal Medicine, Faculty of Medicine, Prince of Songkla University, Hat Yai, Songkhla 90110 Thailand; Department of Internal Medicine, Faculty of Medicine, Prince of Songkla University, Hat Yai, Songkhla 90110 Thailand

**Keywords:** Prealbumin, C-reactive protein, Egg white protein powder, Casein protein

## Abstract

**Background:**

Protein deficiency is a major problem in critically ill patients. Egg white powder recently became a standard additive for protein supplementation in our unit. However, clinical data are not available to support egg white powder supplementation compared to standard protein casein supplementation. This study aimed to determine the change of serum prealbumin (PAB) levels of egg white powder compared to casein additive in standard enteral feeding in critically ill patients with respiratory failure.

**Methods:**

A prospective double-blind, randomized, non-inferiority study was conducted in patients with acute respiratory failure in the medical intensive care unit and respiratory care unit. These patients randomly received 1500 kcal/day of enteral nutrition support with 40 g/day of protein additives by either egg white protein powder or casein protein for 7 days. The serum PAB and C-reactive protein (CRP) levels were measured on days 1, 3, 5, and 7. Repeated-measures ANOVA determined the group effects displayed by serum PAB and CRP levels. *p* values <0.05 were considered statistically significant.

**Results:**

Thirty-four patients were in two groups: 17 in the casein protein group and 17 in the egg white powder group. The clinical characteristics, baseline nutritional status, and biochemistries were not significantly different between the groups. No statistically significant differences were seen in the serum PAB and serum CRP levels between the two groups. The average mean ± SEM difference of serum PAB level between the groups was 2.3 ± 2.5 mg% (*p* = 0.58).

**Conclusions:**

The levels of PAB between the egg white protein additive and casein protein additive were not significantly different and less than the non-inferior margin.

**Trial registration:**

Thai Clinical Trials Registry TCTR20160126002

## Background

Critically ill patients typically develop a catabolic stress state that results in a systemic inflammatory response. This response is coupled with complications of increased infectious morbidity, multiorgan dysfunction, prolonged hospitalization, and disproportionate mortality. Delivering early enteral nutrition support is a proactive therapeutic strategy that can reduce disease severity and diminish complications. This also decreased the length of stay in the intensive care unit (ICU) [1, 2].

In the critical care setting, protein appears to be the most important macronutrient for wound healing, optimizing immunological function, and maintaining lean body mass [3, 4]. However, the common enteral formulas do not meet the protein requirements that are proportionately higher than energy requirements for most critically ill patients [5]. According to the American Society for Parenteral and Enteral Nutrition (ASPEN) guidelines [6], the protein requirement in a critically ill adult patient is in the range of 1.2–2.0 g/kg/day or the presumptive non-protein calories to nitrogen ratio (NPC:N ratio) is about 70:1 to 100:1. Unfortunately, the current common standard enteral formulas have a high NPC:N ratio. Therefore, additional protein supplements are suggested to provide an adequate amount of protein. The European Society of Parenteral and Enteral Nutrition (ESPEN) guidelines suggest that whole-protein formulas, such as milk protein, egg white protein, and soy protein, are among the most appropriate supplementations in critically ill patients in the amount of 1.2–2.0 g/kg/day which depends on the severity of disease [7].

Cow’s milk protein or casein protein was the standard protein supplement in our hospital for several years, but the availability of the product is limited and the cost is high. As a result, egg white protein powder, which is used normally in the food industry, was adopted as the standard additional protein supplementation in our unit because of good availability and lower cost compared to other types of protein additives. Furthermore, given that the levels of biological value (BV) and protein efficiency ratio (PER) of egg white protein are similar to casein protein, the benefits of egg white protein may be similar to the casein protein additive [8–11].

Unfortunately, there is still a lack of clinical data to confirm the benefits of egg white powder supplementation compared to the standard additive proteins, such as whey protein or casein in the critical illness setting. Therefore, we hypothesized that egg white powder supplementation may provide a level of serum prealbumin (PAB) which is not inferior to casein protein supplementation in critically ill patients.

The objective of this study was to determine the change of the serum PAB levels between an egg white powder group and a casein protein additive group in a standard enteral feeding formula in critically ill patients with acute respiratory failure.

## Methods

### Study design and population

A prospective double-blind, randomized non-inferiority study was performed between June 2013 and December 2014 in Songklanagarind Hospital which is a tertiary care hospital with a 10-bed medical ICU and a 16-bed respiratory care unit (RCU). The protocol of this study was approved by the Faculty of Medicine, Prince of Songkla University Institutional Review Board (REC 56-393-14-1). The study protocol was registered in the Thai Clinical Trials Registry (TCTR) and the identification number is TCTR20160126002. Written or oral informed consent was given by the patients or next of kin. Eligibility for the study included patients ≥18 years old with acute respiratory failure which required invasive mechanical ventilation and the initiation of enteral nutrition within 48 h after admission. Exclusion criteria were an expected time of less than five consecutive days for nasogastric tube feeding, patient required top-up parenteral nutrition, morbid obesity (body mass index (BMI) >30 kg/m^2^), renal failure that required renal replacement therapy, hepatic dysfunction (total bilirubin >3 mg%), immune suppressive conditions (e.g., AIDS, chronic steroid use >2 weeks or on immunosuppressive drugs), patients with malignancies who were receiving chemotherapy or radiation therapy, pregnancy, and those with a history of egg white allergies.

### Nutritional management

Nutren Optimum® (Nestlé Nutrition Company, Switzerland) is the standard enteral formula in our unit which contains 1 kcal/mL, protein content of 40 g/1000 kcal, and has an NPC:N ratio of 120:1. The caloric supplement was fixed at 1500 kcal/day with a top-up fixed level of 40 g/day for the additional protein supplementation. Tube feeding began within 48 h after admission and continued for seven consecutive days via continuous infusion pumps or bolus feeding. According to the unit enteral feeding protocol, the caloric supplementation was increased gradually to at least 80 % of the caloric goal within 24–48 h. Metoclopramide, which is a prokinetic agent, was administered intravenously to improve gastric emptying in the patients who had a gastric residual volume greater than 250 mL. The patients were randomly assigned by a computer-generated randomization sequence into two groups of additional protein supplementation. The group assignments were double-blinded by code protein A and protein B. The codes were opened by the investigators after finishing all treatments.

### Data resources and manipulation

Demographics as well as clinical characteristics collected were sex, age, primary diagnosis, APACHE II score, daily and accumulative fluid balance, and vasopressor used. Baseline nutritional status, including BMI, ideal body weight, estimated energy requirement (25 kcal/kg/day), and estimated protein requirement (1.5 g/kg/day) were recorded. Serum PAB was the surrogate of adequate protein energy supplement in this study which is normally used in conjunction with C-reactive protein (CRP) to assess whether the changes in PAB represent adequate protein caloric support or changes in inflammation. The baseline blood chemistries (i.e., serum albumin, BUN, creatinine, liver function test, and zinc level) were also recorded before the beginning of the first day of enteral feeding.

### Outcome variables measurement

The primary endpoint of the study was the change of serum PAB and serum CRP levels in 7 days of nutrition supplementation. Blood samples for PAB and CRP levels were consecutively collected on days 1, 3, 5, and 7 of enteric feeding.

### Statistical analysis

In this non-inferiority study, we postulated that serum PAB levels in the egg white protein group would be non-inferior to the casein protein group by a presumptive non-inferior margin of 5 mg%. We calculated that 17 patients per additive protein group would provide a minimum power of 80 % to determine the non-inferior margin of a 5 mg% using a one-sided *α* of 0.05. Continuous data were expressed as mean and standard error of the mean (SEM). Categorical data were expressed as number and percentage. An independent *t* test was used to compare continuous demographic variables for each treatment group. Fisher’s exact test was used to compare category demographic variables for each treatment group. Repeated-measures ANOVA was used to determine the group effects displayed by the serum PAB and CRP levels. A post hoc analysis of the linear regression model was performed to determine the changes in PAB reflective of adequate caloric and protein support or changes in inflammatory responses. The results were considered statistically significant when the *p* value was less than 0.05. Statistical analysis was computed by SPSS® statistical package version 12.

## Results

### Patient characteristics

Of the 42 patients enrolled, eight patients were excluded: four in the casein protein group and four in the egg white powder group. The excluded cases were one case in the casein protein group that had an expected period of less than 5 days of enteral feeding, one case in the egg white powder group that had a change in parenteral nutrition support, one case in the egg white powder group that required renal replacement therapy, and five cases of death (three in the casein protein group and two in the egg white powder group).

**Fig. 1:**
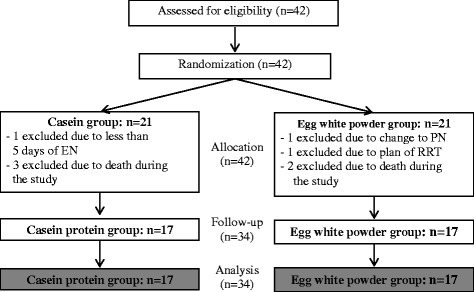
Study overview

Thirty-four patients were eventually obtained for per-protocol analysis (17 in the casein protein group and 17 in the egg white powder group) (Fig. 1). Demographics, clinical characteristics, baseline nutrition assessments, and biochemistry data in the two groups were considered homogeneous (Table 1). The majority of primary diagnoses were respiratory, cardiac, and neurological problems. One patient in the casein protein group was complicated by acute respiratory distress syndrome. Two cases were diagnosed as sepsis before recruitment into the study.

**Table 1:** Demographic, clinical characteristics, baseline nutritional status, and biochemistries in the two groups

Characteristics	Casein group (*n* = 17)	Egg white powder group (*n* = 17)	*p* value
Male sex	52.9 % (9/17)	47.1 % (8/17)	0.73
Age (year)	73.2 ± 3.7	70.3 ± 3.8	0.59
Primary diagnosis (%)			0.31
Respiratory causes	76.5 % (13/17)	58.8 % (10/17)	
Cardiovascular causes	17.7 % (3/17)	11.8 % (2/17)	
Neurological causes	5.9 % (1/17)	17.7 % (3/17)	
Sepsis/septic shock	0	11.8 % (2/17)	
Presence of ARDS (%)	5.9 % (1/17)	0 % (0/17)	0.30
APACHE II score	17.9 ± 1.0	18.6 ± 1.1	0.67
Norepinephrine dose (μg/kg/min)	0.008 ± 0.006	0.024 ± 0.011	0.21
Height (cm)	159.4 ± 2.3	155.9 ± 2.4	0.31
Ideal body weight (kg)	54.2 ± 2.5	50.8 ± 2.7	0.36
BMI (kg/m^2^)	21.3 ± 0.76	21.8 ± 0.9	0.70
Estimated energy requirement (kcal/day)	1626.4 ± 73.7	1523.7 ± 81.9	0.36
Estimated protein requirement (g/day)	81.3 ± 3.7	76.2 ± 4.1	0.36
Average accumulative fluid balance (mL)	4406.9 ± 546.6	6296.1 ± 1420.9	0.20
Average caloric intake (kcal/day)	1402.3 ± 34.7	1453.1 ± 24.3	0.25
Average protein intake (g/day)	89.3 ± 2.6	93.6 ± 1.4	0.17
Average percentage of caloric intake/caloric goal (%)	93.5 ± 2.3	96.9 ± 1.6	0.26
Average gram protein intake (g/kg/day)	1.7 ± 0.1	1.9 ± 0.1	0.19
CRP (mg%)	7.2 ± 1.4	7.3 ± 1.3	0.96
Albumin (g%)	2.7 ± 0.2	2.9 ± 0.1	0.48
Prealbumin (mg%)	13.7 ± 1.7	11.1 ± 1.5	0.25
BUN (mg%)	33.6 ± 5.3	33.8 ± 5.5	0.98
Creatinine (mg%)	1.3 ± 0.2	1.2 ± 0.2	0.91
Total bilirubin (mg%)	0.4 ± 0.1	0.7 ± 0.2	0.12
AST (U/L)	41.3 ± 4.2	65.2 ± 10.8	0.06
ALT (U/L)	34.3 ± 8.9	59.7 ± 19.5	0.27
Zinc level (mg/L)	0.5 ± 0.1	0.5 ± 0.1	0.21

All continuous data are expressed as mean ± SEM. All categorical data are expressed as percentage.

*ARDS* acute respiratory distress syndrome, *APACHE II* acute physiology and chronic health evaluation, *BMI* body mass index, *BUN* blood urea nitrogen, *AST* aspartate aminotransferase, *ALT* alanine aminotransferase

According to the severity of disease, the APACHE II scores in both groups were classified as moderately severe illness concomitant with a significantly high level of baseline serum CRP. Average accumulative fluid balances, average daily dose of norepinephrine, and average caloric and protein intake were not significantly different between the two groups. The average BMI was in the normal range. The daily average fluid balance and daily average norepinephrine requirement in egg white protein group were higher than those in the casein protein group, but they were not statistically significant (Table 2).

**Table 2:** Average daily fluid balance and average daily dose of norepinephrine between the two groups

Day	Casein protein group (*n* = 17)	Egg white powder group (*n* = 17)	*p* value
Average daily fluid balance (mL/day)			
Day 1	773.6 ± 151.2	1291.7 ± 332.1	0.17
Day 2	758.2 ± 155.5	766 ± 232.1	0.98
Day 3	736.3 ± 141.8	1039.7 ± 285.9	0.35
Day 4	527.4 ± 190.4	641.5 ± 229.7	0.71
Day 5	790.3 ± 157.7	872.4 ± 216.1	0.76
Day 6	435.4 ± 174.8	754.6 ± 281.3	0.33
Day 7	385.8 ± 157.1	559.2 ± 215.7	0.51
Total fluid balance (mL)	4406.9 ± 546.6	6296.1 ± 1420.9	0.20
Average daily dose of norepinephrine (μg/kg/min)			
Day 1	0.008 ± 0.006	0.025 ± 0.011	0.21
Day 2	0.004 ± 0.004	0.002 ± 0.002	0.82
Day 3	0.005 ± 0.005	0.008 ± 0.006	0.72
Day 4	0.008 ± 0.005	0.159 ± 0.008	0.47
Day 5	0.019 ± 0.015	0.012 ± 0.012	0.75
Day 6	0.034 ± 0.03	0.0 ± 0.0	0.27
Day 7	0.034 ± 0.03	0.0 ± 0.0	0.27

All data are expressed as mean ± SEM

In both groups, the actual caloric intake as well as protein intake achieved 80 % of target within 72 h after enteral feeding initiation. The mean percentages ± SEM of the actual caloric intake during the study period were 93.5 ± 2.3 % in the casein protein supplementation group and 96.9 ± 1.6 % in the egg white protein powder supplementation group. In addition, the mean ± SEM actual protein intakes in the casein protein additive and egg white protein additive groups were 1.7 ± 0.1 and 1.9 ± 0.1 g/kg/day, respectively.

### Changes of serum PAB and CRP levels

The daily changes of the serum PAB and CRP levels in each group and between the groups are shown in Tables 3 and 4, respectively. The changes of serum PAB and CRP in the casein protein additive group were not significantly different from day 1 to day 7, but the serum CRP levels in the egg white protein additive group decreased significantly from day 1 to day 7.

**Table 3:** Serum PAB levels between the two groups on days 1, 3, 5, and 7

Day	Casein protein group (*n* = 17)		Egg white powder group (*n* = 17)		*p* value (between groups)
	Biochemical values	*p* value (compared to day 1)	Biochemical values	*p* value (compared to day 1)	
Serum PAB level (mg %)					
Day 1	13.7 ± 1.7		11.1 ± 1.5		0.25
Day 3	14.3 ± 1.8	0.29	11.4 ± 1.6	0.52	0.23
Day 5	14.2 ± 1.9	0.64	11.8 ± 1.6	0.46	0.33
Day 7	15.8 ± 1.9	0.13	14.6 ± 2.1	0.21	0.70

All data are expressed as mean ± SEM; repeated-measures ANOVA model was applied

**Table 4:** Serum CRP levels between the two groups on days 1, 3, 5, and 7

Day	Casein protein group (*n* = 17)		Egg white powder group (*n* = 17)		*p* value (between groups)
	Biochemical values	*p* value (compared to day 1)	Biochemical values	*p* value (compared to day 1)	
CRP (mg %)					
Day 1	7.2 ± 1.4		7.3 ± 1.3		0.96
Day 3	5.2 ± 0.9^a^	0.03	5.6 ± 1.1	0.16	0.74
Day 5	6.0 ± 1.5	0.40	5.0 ± 1.1	0.08	0.61
Day 7	6.6 ± 1.6	0.66	3.5 ± 0.7^a^	0.01	0.09

All data are expressed as mean ± SEM; repeated-measures ANOVA model was applied

^a^Significantly different from day 1 within the group comparison (*p* value <0.05)

Although the serum PAB essentially increased in both groups, no statistically significant difference in the serum PAB between the two groups was seen on days 1, 3, 5, and 7 (Table 3). The average mean ± SEM difference of the serum PAB between the two groups was 2.3 ± 2.5 mg%, which was less than the postulated non-inferior margin of the study (*p* = 0.58). In addition, the serum CRP indicated a decreasing trend in both groups but no statistically significant difference between the groups was found (Table 4).

We performed a post hoc analysis of the linear regression model to determine the changes in PAB reflective of adequate caloric and protein support or changes in inflammatory responses. Apparently, the change in serum CRP was the only independent factor to predict changes in the serum PAB in all cases (adjusted odds ratio (OR), −0.53, 95 % CI −0.96 to −0.23, *p* = 0.002) and in the casein protein additive group (adjusted OR −0.87, 95 % CI −1.16 to −0.60, *p* < 0.001). No correlation was found to predict changes in PAB from the change in serum CRP in the egg white protein group (adjusted OR −0.38, 95 % CI −1.53 to 0.46, *p* = 0.26)*.* Furthermore, changes in both caloric and protein intakes were not independent factors to predict changes in the serum PAB (adjusted OR 0.47, 95 % CI −0.04 to 0.02, *p* = 0.18 and adjusted OR −0.39, 95 % CI −0.26 to 0.03, *p* = 0.26).

## Discussion

A caloric goal of 25 kcal/kg/day and a protein goal in the range of 1.2–2.0 g/kg/day were suggested according to the recent standard recommendations of caloric and protein intake in critically ill circumstances [6]. Top-up proteins, for example, milk protein, egg white protein, and soy protein, are among the most appropriate supplementations in critically ill patients according to the recent ESPEN guideline [7].

Some experts suggested that the biological value (BV) of protein and protein efficiency ratio (PER) be considered to select the appropriate type of protein supplementation [9, 12, 13]. Biological value of protein is a measure of the proportion of absorbed protein that can be used in protein synthesis in the cells of the human body. When a protein contains the amount of essential amino acids equivalent to requirement, the BV of protein is significantly high [14]. In addition, the PER is the ratio of grams of body weight gain to the grams of protein consumed [9]. Animal protein apparently gives a higher BV and PER than plant protein. Whey protein is classified as the highest in both BV and PER, followed by whole egg protein, casein protein, and egg white protein [9, 11]. However, the clinical evidence of comparing specific types of protein is limited. In a small randomized study of whey protein and casein protein supplement in acute stroke patients, the whey protein additive may decrease inflammation and increase antioxidant levels in patients with acute stroke compared to casein protein additive [15].

In developing countries, protein supplements are limited in supply and they are costly. Casein protein additive was used regularly in our unit as a standard protein additive for a period of time, but the availability was limited and the cost was high. For this reason, protein supplementation in critically ill patients was possibly inadequate. Egg white protein powder, which is normally used in the food industry, is widely available, is less costly, and possibly gives a similar BV and PER to casein protein. As a result, this type of protein additive has been suggested as a substitute for casein protein additive in our unit. This study was conducted to determine the effect of serum protein marker change between egg white protein additive and casein additive in critically ill patients by the non-inferiority fashion.

The results of this study demonstrated that the increment of serum PAB level in egg white protein additive was not inferior to the casein protein additive group (*p* = 0.58) with an average mean ± SEM difference of 2.3 ± 2.5 mg%. However, the increments of PAB within both groups were not different from day 1 to day 7. The low increments in the serum PAB levels were lower than the physiological expectations which were possibly affected by the catabolic stress state in those with critical illnesses. From our findings, the positive accumulative daily fluid balance and the amount of norepinephrine requirement indicated incomplete resolution of catabolic responses during the study period. Furthermore, the large variability of the pathology of acute respiratory failure in this study may influence the alteration of serum PAB. The small sample sizes of each pathology may lead to an unconcluded definite outcome of this trial.

From a recent study, an 80 % of caloric intake to caloric goal demonstrated significantly better outcomes that included ICU morbidity and mortality [16]. The adequacy of caloric and protein support in this study was evidenced by the average percentage of caloric intake to caloric goal greater than 90 % in both groups, and the range of protein intake in both groups was 1.7–1.9 g/kg/day. Therefore, the influence of serum protein markers by the amount of protein calories could be demonstrated.

PAB is normally suggested as a marker of adequate caloric and protein support in preference to other serum protein markers such as serum albumin and serum transferrin [17]. Moreover, the PAB level correlates well with clinical outcomes, has a short half-life of about 2.5 days, is less affected by liver disease, and is not affected by hydration status. In patients who receive adequate caloric and protein supplementation, the PAB level should rise by 2 mg% per day and then return to normal within 4–8 days [18]. However, numerous factors may interfere with the level of serum PAB, for example, severe renal failure, corticosteroid use, oral contraception use, significant hyperglycemia, and hemodialysis. Furthermore, the level of serum PAB may be significantly reduced by the reduction of PAB synthesis in acute inflammatory state [12, 19]. Therefore, CRP is the most popular inflammatory protein marker generally used in conjunction with PAB to determine the changes in PAB reflective of adequate caloric support or changes in inflammatory responses.

CRP is a common protein marker for inflammation which rapidly changes in the systemic inflammatory status in both acute and chronic responses. The half-life of CRP is approximately 19 h and can change rapidly with the level of inflammatory response. However, the limitations of using CRP to determine the severity and stage of inflammatory responses were reported [20–22].

From a recent retrospective study, the change in serum CRP was the only independent factor of serum PAB change rather than the change of nutrition intake. Therefore, the serum PAB may be influenced by inflammatory responses rather than adequate caloric and protein support [23]. However, the caloric and protein intake in that retrospective study was only 50–60 % of caloric goal, which was significantly lower than our study and could be a limitation to find the correlation.

Our study also demonstrated the improvement of inflammatory markers by egg white protein supplementation. The mean serum CRP level decreased significantly in day 7 compared to day 1 in the egg white powder group, determined by repeated-measures ANOVA (*p* = 0.01). The improvement of the oxidative stress may theoretically improve the balance of protein anabolism/catabolism with comprehensive benefits in critically ill patients. The additional protein supplementation, especially cysteine-rich egg white protein, increased the amounts of substrate for the synthesis of glutathione [24]. The increment of glutathione, which is a natural antioxidant, will attenuate the severity of inflammatory responses and result in a lower serum CRP level. However, the decrement of serum CRP in our study could not be distinguished between the recoveries of illness from the effect of nutritional therapy or the specific type of protein. The correlation of the egg white protein or other specific types of protein additives and the alteration of inflammatory markers may require a larger study.

To determine the changes in serum PAB reflective of adequate caloric and protein support or changes in inflammatory responses, the post hoc analysis by the linear regression model indicated the changes of caloric and protein support were not predictors for serum PAB changes. In addition, the change of serum CRP as a surrogate of inflammation may not correlate totally to the change of serum PAB because this correlation was found only in the casein group and not in the egg white protein group. There are some other explanations. First, this study was not designed to determine the regression analysis, so the sample sizing may not be adequate to detect the correlation. Second, other factors which could not be measured in this study due to limited resources were the blood level interleukin, tumor necrosis factor, and other inflammatory cytokines which may influence the changes of PAB over time.

Although previous studies suggested PAB as a surrogate of adequate caloric and protein intake in critically ill patients [17, 25, 26], our study may contradict that suggestion. Other serum protein markers, such as serum free amino acids [27, 28] or the measurement of muscle mass and function [29, 30], could be used as a surrogate in further protein supplement studies in critically ill patients. Although, improvements of biochemistry results were shown in this study, the clinical outcomes still need to be confirmed.

Apart from those limitations, our study was the first prospective double-blinded randomized control study to compare the benefits of two types of protein supplementation which included casein protein and egg white protein in a critically ill cohort. The inclusion and exclusion criteria were rigid which diminished bias and confounding factors for changes in serum protein markers by disease conditions and treatment modalities. This study was intentionally conducted to solve the real-life uncertainties that occur during daily clinical practice in caring for critically ill patients. The results of this type of study may ensure the ongoing process of care and might be applicable in regions of limited resources.

Although the serum PAB may not be an appropriate serum protein marker to determine the benefit of the type of protein supplementation, an adequate dosage of either casein protein or egg white protein may be beneficial to maintain the level of specific serum protein markers in critically ill patients.

## Conclusions

Although the levels of PAB between the egg white protein additive and casein protein additive were not significantly different and less than the non-inferior margin of 5 mg%, this result cannot be totally concluded because no significant correlation in the change of caloric or protein intake to predict a change in serum PAB was detected. A further study to determine another type of serum protein marker or muscular functional test needs to be performed.
